# Improved bootstrap $\bar{X}$ control chart for non-normally distributed data

**DOI:** 10.1016/j.mex.2025.103190

**Published:** 2025-01-30

**Authors:** Sukma Adi Perdana, Muhammad Mashuri, Muhammad Ahsan

**Affiliations:** a Department of Statistics, Faculty of Science and Data Analytics, Institut Teknologi Sepuluh Nopember, Indonesia; b STAIN Sultan Abdurrahman Kepulauan Riau, Bintan, Indonesia

**Keywords:** Control charts, Bootstrap control chart, Non-normal data, Improved bootstrap control chart, Improved Bootstrap Control Chart

## Abstract

This article proposes new method to improve the performance of bootstrap control chart for non-normal data. Bootstrap control charts for monitoring data require attention because the average run length (ARL) results of the bootstrap control charts can be less accurate and lacks stability. To deal with this issue, $\bar{X}$ control chart from non-normal distribution was proposed using a novel improved Bootstrap method. The control limit is constructed using improved bootstrap to ensure that the UCL and LCL obtained actually give the desired ARL_0_ value. Comparison of performance is studied among the Bajgier bootstrap control chart, Liu Tang Bootstrap, and their Improved bootstrap control chart. The result show that the proposed bootstrap control chart has better accuracy than its classic method. Key points:

• A novel improved bootstrap method is proposed to deal with the ARL results of the bootstrap control charts that can vary.
• The new $\bar{X}$ Control Chart from non-normal distribution was constructed using a novel improved bootstrap method.

## Graphical abstract

**Figure:**
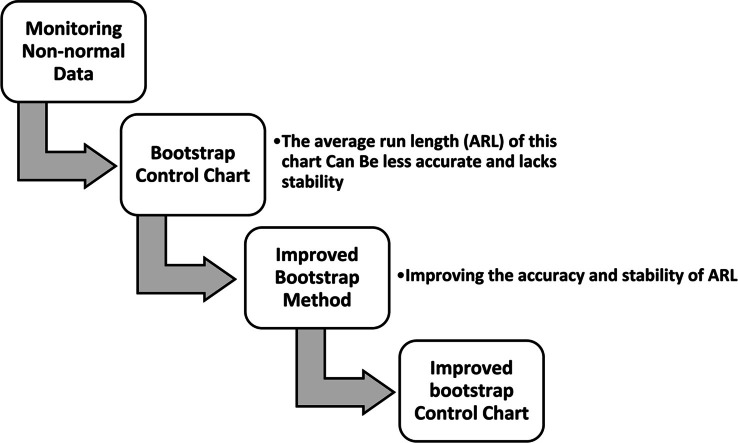

Specifications table

**Table:** 

Subject area:	Mathematics and Statistics
More specific subject area:	Statistics; Statistical Process Control
Name of your method:	Improved Bootstrap Control Chart
Name and reference of original method:	*Liu, R. Y., & Tang, J. (1996). Control Charts for Dependent and Independent Measurements Based on Bootstrap Methods. Journal of the American Statistical Association, 91(436), 1694–1700.* https://doi.org/10.1080/01621459.1996.10476740
Resource availability:	Data will be made available on request.

## Background

In constructing $\bar{X}$ Control Chart, assumed distribution plays an important role in ensuring that the control chart works properly. Shewhart control charts are highly effective for mean monitoring in normally distributed populations. However, for non-normally distributed data, the accuracy of the control chart in identifying assignable causes is not as good as that in a normal distribution.

In practice, the data that is monitored sometimes comes from a non-normal distribution. 235 quality control implementations were found to frequently violate the assumptions of normality and independence [1]. The consequence of not fulfilling these assumptions is an error within the control limits. The presence of abnormalities significantly affects control chart performance. It was showed that departures from normality impact the error probabilities and average run-length properties significantly [2]. It was stated that incorrect decisions could result from using normal distribution-based control chart methods on data that illustrate a non-normal quality characteristic distribution [3].

Efforts are being made to alleviate the effects of non-normality in the control chart and maintain accurate process monitoring. Aichouni et al. employed Johnson's family of distributions to design control charts and used this method in ready mixed concrete plants with skewed non-normal process distributions [4]. Schoonhoven & Does [5] adopted a unique design scheme approach for $\bar{X}$ control charts with non-normal distributions. For moderate sample sizes (20 subgroups of size 4–10), Schoonhoven & Does [5] examined the performance of different standard deviation estimators in control charts under non-normality. Karagöz & Hamurkaroğlu [6] utilized classic, weighted variance, weighted standard deviation, and skewness correction methods when constructing control charts for Weibull, Gamma, and lognormal distributions.

An alternative approach to handle non-normality in control charts is through the bootstrap method. Specifically, the nonparametric bootstrap can be used to determine a statistic's sample distribution. Several articles proposed employing nonparametric bootstrap for statistical process monitoring. An in-depth examination of bootstrap techniques for statistical process monitoring can be found by interested readers in [[7], [8], [9], [10], [11], [12]]. According to Jones and Woodall [13], The variability of average run length (ARL) in bootstrap control charts is considerable, particularly in the estimation of a skewed distribution's extreme tails [13]. Therefore, to obtain a more reliable and superior ARL, It is necessary to improve the bootstrap approach.

This study presents an improved bootstrap control chart for data or samples from non-normally distributed populations. The bootstrap control chart algorithm is modified in the proposed method to enhance control chart accuracy. To evaluate the effectiveness of the proposed algorithm, the ARL of the Bajgier bootstrap control chart [13], the Liu Tang bootstrap control chart [9], and the improved bootstrap control chart were evaluated using simulation.

## Method details

### Shewhart $\bar{X}$ control chart

By following the central limit theorem with parameters

$$\mu_{\bar{X}}=\theta \;and\;\sigma_{\bar{X}}^{2}=\frac{\theta^{2}}{n}\;so\;\sigma_{\bar{X}}=\frac{\theta }{\sqrt{n}}$$

So, with α=0.027, According to [14], Shewhart $\bar{X}$ control chart can be described as follows:

$$UCL=\theta +3.\frac{\theta }{\sqrt{n}}$$

CentreLine=θ

$$LCL=\theta -3.\frac{\theta }{\sqrt{n}}$$

### Bajgier bootstrap control chart

Instead of a Shewhart control chart, Bajgier suggested employing a nonparametric bootstrap control chart. The Bajgier bootstrap control chart is constructed as follows [7,13].

1. Collect k subgroups of n sizes, yielding n.k observations in total.
2. From n.k observations, select a size-$n_{b}$ random sample with replacement. This sample, $x_{1}^{*}$, $x_{2}^{*}$…,$x_{n_{b}}^{*}$ is a bootstrap sample.
3. Calculate the sample mean ${\bar{x}}_{i}$ from each bootstrap sample mean $x_{1}^{*}$, $x_{2}^{*}$…,$x_{n_{b}}^{*}$.
4. Proceed through steps 2–3 multiple times, let's say B times.
5. Sort the sample mean B bootstrap ${\bar{x}}_{1}^{*}$, ${\bar{x}}_{2}^{*}$,…, ${\bar{x}}_{B}^{*}$.
6. Determine the bootstrap lower control limit (LCL) or $LCL_{j}^{*}$ which is ${\left(\alpha \;/\;2\right)}^{th}$ percentile of the ‘B’ bootstrap sample means ${\bar{x}}_{1}^{*}$, ${\bar{x}}_{2}^{*}$,…, ${\bar{x}}_{B}^{*}$.
7. Determine the bootstrap upper control limit (UCL) or $UCL_{j}^{*}$ which is ${\left(1-\alpha \;/\;2\right)}^{th}$ percentile of the ‘B’ bootstrap sample means ${\bar{x}}_{1}^{*}$, ${\bar{x}}_{2}^{*}$,…, ${\bar{x}}_{B}^{*}.$.

### Liu tang bootstrap control chart

The Liu Tang bootstrap control chart is constructed through the following steps [9,13].

1. Collect k subgroups of n sizes, yielding n.k observations in total.
2. Calculate ${\bar{\bar{X}}}_{N}=\frac{1}{N}\sum_{i=1}^{k}\sum_{j=1}^{n}x_{ij}$.
3. From n.k observations, select a size-n random sample with replacement. This sample, $x_{1}^{*}$, $x_{2}^{*}$…,$x_{n}^{*}$ is a bootstrap sample.
4. Calculate the sample mean ${\bar{x}}^{*}=\frac{1}{n}\sum_{i=1}^{n}x_{i}^{*}$ and $t^{*}=\sqrt{n}\left({\bar{x}}^{*}-{\bar{\bar{X}}}_{N}\right)$ from the bootstrap sample drawn in step 3.
5. Proceed through steps 3–4 multiple times, let's say B times.
6. Sort the sample mean B bootstrap $t_{1}^{*}$, $t_{2}^{*}$,…, $t_{B}^{*}$.
7. Determine $t_{\alpha \;/\;2}^{*}$ which is ${\left(\alpha \;/\;2\right)}^{th}$ percentile of the ‘B’ bootstrap sample means $t_{1}^{*}$, $t_{2}^{*}$,…, $t_{B}^{*}$.
8. Determine $t_{1-\alpha \;/\;2}^{*}$ which is ${\left(1-\alpha \;/\;2\right)}^{th}$ percentile of the ‘B’ bootstrap sample means $t_{1}^{*}$, $t_{2}^{*}$,…, $t_{B}^{*}$.
9. Calculate the LCL and UCL using
  $$LCL={\bar{\bar{X}}}_{N}+\frac{t_{\alpha \;/\;2}^{*}}{\sqrt{n}}$$

And

$$UCL={\bar{\bar{X}}}_{N}+\frac{t_{1-\alpha \;/\;2}^{*}}{\sqrt{n}}$$

### The improved bootstrap control chart

The improved control chart's main principle is to ensure that the UCL and LCL obtained actually give an ARL_0_ value of 370 for α=0.0027**.** This is accomplished by executing the optimal point search for both UCL and LCL, resulting in the desired $\text{AR}L_{0}$ value. The bisection algorithm [[15], [16], [17], [18]] involving δ and ε inspires the optimization process of the ARL value. The principle of this process is that the LCL and UCL values are shifted by δ to obtain LCL and UCL values that provide the desired $\text{AR}L_{0}$ value with an error bounded by ε. The selection of δ and ε in the simulation depends on the magnitude of the control limit values to achieve the best optimization time. As explained below, the improved algorithm developed is subsequently added to the Bagjer Bootstrap Control Chart and Liu Tang Bootstrap Control Chart algorithms.

### Improved bajgier bootstrap control chart

Based on Bajgier Bootstrap control chart, the improved bootstrap control chart for α=0.0027 is constructed as follows:

1. Collect k subgroups of n sizes, yielding n.k observations in total.
2. From n.k observations, select a size-$n_{b}$ random sample with replacement. This sample, $x_{1}^{*}$, $x_{2}^{*}$…,$x_{n_{b}}^{*}$ is a bootstrap sample.
3. Calculate the sample mean ${\bar{x}}_{i}$ from each bootstrap sample mean $x_{1}^{*}$, $x_{2}^{*}$…,$x_{n_{b}}^{*}$.
4. Proceed through steps 2–3 multiple times, let's say B times.
5. Sort the sample mean B bootstrap ${\bar{x}}_{1}^{*}$, ${\bar{x}}_{2}^{*}$,…, ${\bar{x}}_{B}^{*}$.
6. Determine the bootstrap lower control limit (LCL) or $LCL_{j}^{*}$ which is ${\left(\alpha \;/\;2\right)}^{th}$ percentile of the ‘B’ bootstrap sample means ${\bar{x}}_{1}^{*}$, ${\bar{x}}_{2}^{*}$,…, ${\bar{x}}_{B}^{*}$.
7. Determine the bootstrap upper control limit (UCL) or $UCL_{j}^{*}$ which is ${\left(1-\alpha \;/\;2\right)}^{th}$ percentile of the ‘B’ bootstrap sample means ${\bar{x}}_{1}^{*}$, ${\bar{x}}_{2}^{*}$,…, ${\bar{x}}_{B}^{*}$.
8. Calculate the ARL_0_ value with the LCL and UCL value results of the previous step by using n.k observations from step 1.
9. Specify the value of δ and ε.
10. Specify UCL = UCL + δ, LCL= LCL – δ,and up = 0 if ARL_0_ < 370 Or UCL = UCL – δ, LCL =LCL + δ, and up= 1 if ARL_0_ > 370
11. Calculates the ARL_0_ value with new LCL and UCL by using n.k observations from step 1.
12. Specify δ=δ/2 if $ARL_{0}$ < 370 and up = 1 And specify δ=δ/2 if $ARL_{0}$ > 370 and up = 0.
13. Stops the process if $|ARL_{0}-370|\leq \varepsilon$ and continues the process when $|ARL_{0}-370|>\varepsilon$.
14. Repeat steps 12–15 until $|ARL_{0}-370|\leq \varepsilon$ in order to obtain the best LCL and UCL.

### Improved liu tang bootstrap control chart

Based on Liu Tang Bootstrap control chart, the improved bootstrap control chart for α=0.0027 is constructed as follows:

1. Collect k subgroups of n sizes, yielding n.k observations in total.
2. Calculate ${\bar{\bar{X}}}_{N}=\frac{1}{N}\sum_{i=1}^{k}\sum_{j=1}^{n}x_{ij}$.
3. From n.k observations, select a size-n random sample with replacement. This sample, $x_{1}^{*}$, $x_{2}^{*}$…,$x_{n}^{*}$ is a bootstrap sample.
4. Calculate the sample mean ${\bar{x}}^{*}=\frac{1}{n}\sum_{i=1}^{n}x_{i}^{*}$ and $t^{*}=\sqrt{n}\left({\bar{x}}^{*}-{\bar{\bar{X}}}_{N}\right)$ from the bootstrap sample drawn in step 3.
5. Proceed through steps 3–4 multiple times, let's say B times.
6. Sort the sample mean B bootstrap $t_{1}^{*}$, $t_{2}^{*}$,…, $t_{B}^{*}$.
7. Determine $t_{\alpha \;/\;2}^{*}$ which is ${\left(\alpha \;/\;2\right)}^{th}$ percentile of the ‘B’ bootstrap sample means $t_{1}^{*}$, $t_{2}^{*}$,…, $t_{B}^{*}$.
8. Determine $t_{1-\alpha \;/\;2}^{*}$ which is ${\left(1-\alpha \;/\;2\right)}^{th}$ percentile of the ‘B’ bootstrap sample means $t_{1}^{*}$, $t_{2}^{*}$,…, $t_{B}^{*}$.
9. Calculate the LCL and UCL using
  $$LCL={\bar{\bar{X}}}_{N}+\frac{t_{\alpha \;/\;2}^{*}}{\sqrt{n}}$$
  And
  $$UCL={\bar{\bar{X}}}_{N}+\frac{t_{1-\alpha \;/\;2}^{*}}{\sqrt{n}}$$
10. Calculate the ARL_0_ value with the LCL and UCL value results of the previous step by using n.k observations from step 1.
11. Specify the value of δ and ε.
12. Specify UCL = UCL + δ, LCL= LCL – δ,and up = 0 if ARL_0_ < 370 Or

UCL = UCL – δ, LCL =LCL + δ,and up= 1 if ARL_0_ > 370

13. Calculates the ARL_0_ value with new LCL and UCL by using n.k observations from step 1.
14. Specify δ=δ/2 if $ARL_{0}$ < 370 and up = 1

And specify δ=δ/2 if $ARL_{0}$ > 370 and up = 0.

15. Stops the process if $|ARL_{0}-370|\leq \varepsilon$ and continues the process when $|ARL_{0}-370|>\varepsilon$.
16. Repeat steps 12–15 until $|ARL_{0}-370|\leq \varepsilon$ in order to obtain the best LCL and UCL.

## Method validation

### Simulation

The simulation assessed the proposed bootstrap control chart methods. The performance of Bajgier bootstrap, Liu Tang bootstrap, and their improved bootstrap control charts were examined in terms of the ARL for the in-control situation, commonly referred to as $\text{AR}L_{0}$. Performance evaluation focuses on the average $\text{AR}L_{0}$ value (A$\text{AR}L_{0}$) and the standard deviation of $\text{AR}L_{0}$ (SD$\text{AR}L_{0}$). This is because the estimation of the parameters turns the $\text{AR}L_{0}$ into a random variable. Therefore, assessing performance requires a consideration of both the average ARL value (A$\text{AR}L_{0}$) and the standard deviation of the ARL (SD$\text{AR}L_{0}$) [19,20]. This is important because various practitioners could produce different estimations of the process parameters, which could result in different $\text{AR}L_{0}$ values. As a result, the $\text{AR}L_{0}$ becomes random due to practitioner-to-practitioner variability [21].

Following the study of [22], simulations were performed for normal distribution, heavy-tailed symmetrical distributions such as Student's t distribution, and skewed distributions such as Gamma(5,1). These distributions were selected because of their skewness and heavy-tailed characteristics. The simulations are performed for sample size (n=5, 10, 30) and subgroup size k = 30. The sample size was chosen as n=5 to represent a small sample size, n=10 to represent a medium sample size, and n=30 to represent a large sample size. On the improved bootstrap algorithm, we set δ=1 and ε=5, The values are selected based on the LCL and UCL magnitudes before the improvement process begins. Here, we compare the $\text{AR}L_{0}$ from the Bajgier bootstrap, Liu Tang bootstrap, and their improved bootstrap control chart to see which control chart has the $\text{AR}L_{0}$ value more accurate or closer to the value of 370 for α=0.0027. Following the study of Chen et al [20], The identical simulation was conducted 10,000 times, producing 10,000 $\text{AR}L_{0}$ values. From these, we calculated the mean ARL_0_ (AARL_0_) and standard deviation of ARL_0_ (SDARL_0_). This process allows us to evaluate the accuracy and dispersion of the $\text{AR}L_{0}$ values obtained from the tested method. The AARL_0_ represents the average $\text{AR}L_{0}$ value generated by the method, while the SDARL_0_ indicates the degree of dispersion among the $\text{AR}L_{0}$ values, reflecting how far they spread from their central point, or AARL_0_. The optimal method is the one that yields an AARL_0_ value closest to the desired target of 370 and produces the smallest SDARL0 value.

### Evaluation findings

As explained in the previous section, simulations were conducted to obtain the $\text{AAR}L_{0}$ and $\text{SDAR}L_{0}$ values from the four compared methods, namely the classic Bajgier bootstrap control chart (CBB), the improved Bajgier bootstrap control chart (IBB), the classic Liu Tang Bootstrap control charts (CLB), and the improved Liu Tang bootstrap control chart. (ILB). The better method is the one with an $\text{AAR}L_{0}$ value closer to 370 and a smaller SDARL value. Table 1 shows the $\text{AAR}L_{0}$ values for the four compared bootstrap methods. The first column contains information about the compared methods. In the second, third, and fourth columns, information on the $\text{AAR}L_{0}$ values of the compared methods is presented, where the data is generated from a normal distribution with n subgroups of 5, 10, and 30. The fifth, sixth, and seventh columns provide information on the $\text{AAR}L_{0}$ values derived from a gamma distribution with n subgroups of sizes 5, 10, and 30. Meanwhile, the eighth, ninth, and tenth columns contain information on the $\text{AAR}L_{0}$ values from data from a Student's t distribution.

**Table 1:** Comparison of $\text{AAR}L_{0}$ for all bootstrap control chart methods with α=0.0027.

Bootstrap Methods	Normal Distribution			Gamma Distribution			Student's t distribution		
	5	10	30	5	10	30	5	10	30
CBB	251.069	248.350	238.328	249.516	246.839	239.437	211.827	246.014	236.032
IBB	360.351	356.634	343.087	359.672	357.765	343.222	285.666	352.262	341.462
CLB	250.015	249.741	238.347	249.368	249.238	236.385	210.981	243.227	238.780
ILB	362.412	356.187	340.710	359.288	356.410	342.162	287.242	349.996	342.108

As shown in Table 1, the method that yields the $\text{AAR}L_{0}$ value closest to 370 for data generated from a normal distribution is the ILB method with a percentage of $\text{AAR}L_{0}$ values close to 370 of 97.39 % for n=5, and the IBB method for n=10 and n=30 with a percentage of $\text{AAR}L_{0}$ values close to 370 of 96.39 % and 92.73 % respectively . Meanwhile, the method that produces the $\text{AAR}L_{0}$ value closest to 370 for data from a gamma distribution for n=5, 10, and 30 is the IBB method, with $a\;\text{percentage}\;\text{of}\;\text{AAR}L_{0}\;\text{values}\;\text{close}\;\text{to}\;370\;\text{of}\;97.21░\%,\;96.69░\%,\;\text{and}\;92.76░\%,$ respectively. Next, the method that produces the $\text{AAR}L_{0}$ value closest to 370 for data generated from a student's t distribution is the ILB method for n=5 and n=30 with $a\;\text{percentage}\;\text{of}\;\text{AAR}L_{0}\;\text{values}\;\text{close}\;\text{to}\;370\;\text{of}\;77.63░\%\;\text{and}\;92.46░\%\;$respectively, and the IBB method for n=10 with a percentage of $\text{AAR}L_{0}$ values close to 370 of 95.21 % . From the information in Table 1, it can be seen that the IBB and ILB methods always provide $\text{AAR}L_{0}$ values closer to the expected $\text{AAR}L_{0}$ value of 370 compared to the CBB and CLB methods. Meanwhile, the difference in all $\text{AAR}L_{0}$ values between IBB and ILB does not appear to be significantly different.

Furthermore, to examine the dispersion of $\text{AR}L_{0}$ values, the $\text{SDAR}L_{0}$ values are also presented in Table 2. The first column of Table 2 provides details about the methods being compared. The second, third, and fourth columns display $\text{SDAR}L_{0}$ values for data generated from a normal distribution, organized by subgroups of sizes 5, 10, and 30. The fifth, sixth, and seventh columns provide $\text{SDAR}L_{0}$ values for data from a gamma distribution, also categorized by 5, 10, and 30 subgroups. The eighth, ninth, and tenth columns outline $\text{SDAR}L_{0}$ values for data derived from a student's t-distribution.

**Table 2:** Comparison of $\text{SDAR}L_{0}$ for all bootstrap control chart methods.

Bootstrap Methods	Normal Distribution			Gamma Distribution			Student's t distribution		
	5	10	30	5	10	30	5	10	30
CBB	117.725	117.970	118.818	119.073	118.937	120.210	130.987	123.887	119.791
IBB	71.069	73.226	84.498	83.480	81.558	91.362	148.979	93.916	87.043
CLB	116.388	118.615	119.473	118.821	118.480	118.277	129.814	121.092	120.100
ILB	72.132	72.466	85.633	83.361	80.306	90.543	147.480	93.705	87.620

From the information presented in Table 2, it is evident that the method yielding the lowest $\text{SDAR}L_{0}$ values for data derived from a normal distribution is the IBB method, particularly for sample sizes n=5 and n=30, with $\text{SDAR}L_{0}$ values of 71.069 and 84.498, respectively. For a sample size of n=10, the ILB method achieves the smallest $\text{SDAR}L_{0}$ value of 72.466. Conversely, when analyzing data from a gamma distribution, the ILB method consistently produces the smallest $\text{SDAR}L_{0}$ values across sample sizes n=5, 10, and 30, with values of 83.361, 80.306, and 90.543, respectively. Regarding the Student's t distribution, the CLB method demonstrates the lowest $\text{SDAR}L_{0}$ value for n=5 at 129.814, while the ILB method is optimal for n=10, achieving $\text{SDAR}L_{0}$ value of 93.705. For n=30, the IBB method again provides the smallest $\text{SDAR}L_{0}$ value of 87.043.

Based on the previous explanation, the $\text{AAR}L_{0}$ value is consistently closer to 370 for every improved bootstrap control chart than its classic form. This result indicates that each improved bootstrap control chart is consistently better than its classic form. In addition to looking at the $\text{AAR}L_{0}$ value from the bootstrap control chart, the $\text{SDAR}L_{0}$ value is also evaluated to determine the dispersion of the $\text{AR}L_{0}$ value of the control charts. According to Table 2, the larger the sample size (*n*), the $\text{SDAR}L_{0}$ value of the improved bootstrap will be smaller than the $\text{SDAR}L_{0}$ of the classic form for any bootstrap control chart method. This finding indicates that classic bootstrap control charts exhibit more significant variability than the improved versions. The accuracy evaluation by the $\text{AAR}L_{0}$ value shows that the improved bootstrap control charts have better accuracy than its classic forms. Moreover, the improved bootstrap method exhibits less variability than classic bootstrap control charts.

### Practical scenario

**Fig. 1:**
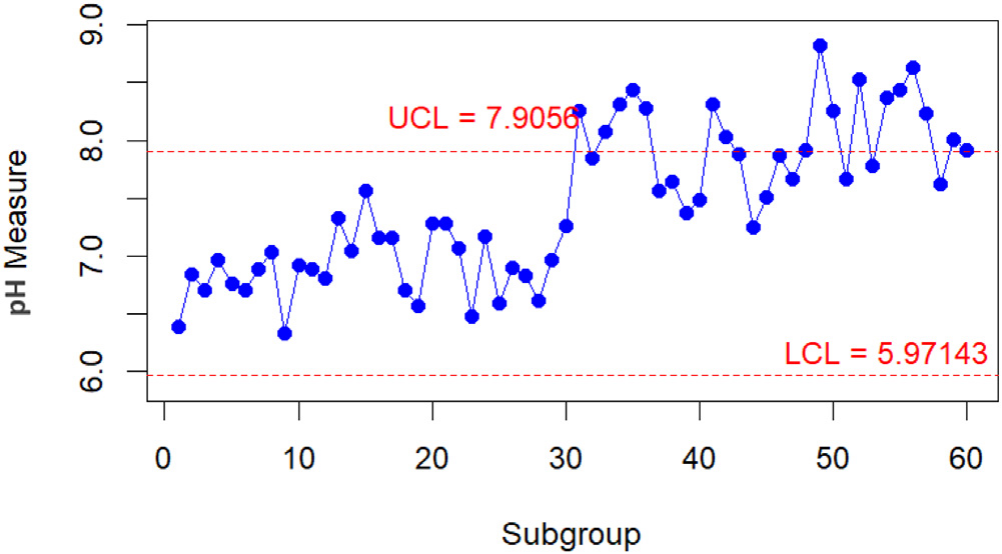
The Bajgier bootstrap control chart.

**Fig. 2:**
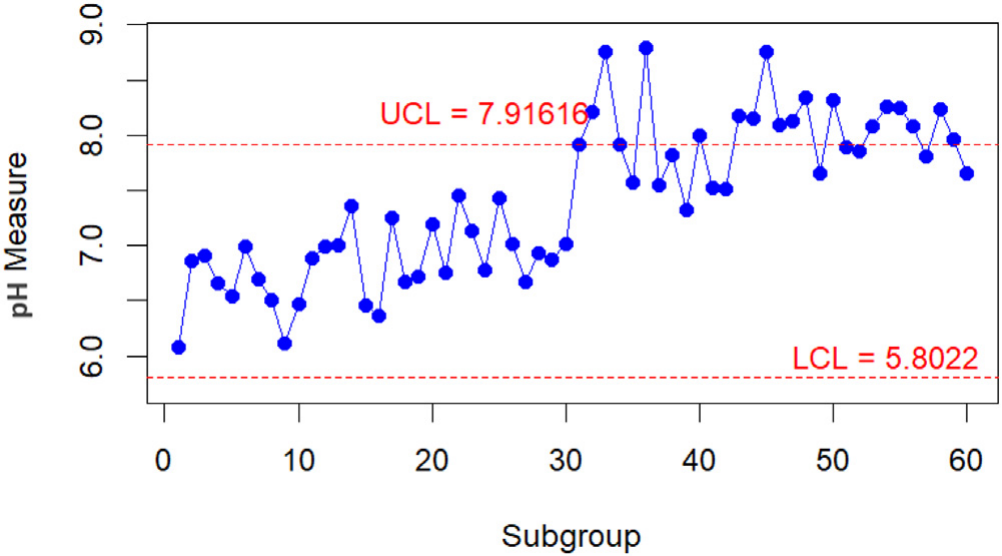
The Liu Tang bootstrap control chart.

**Fig. 3:**
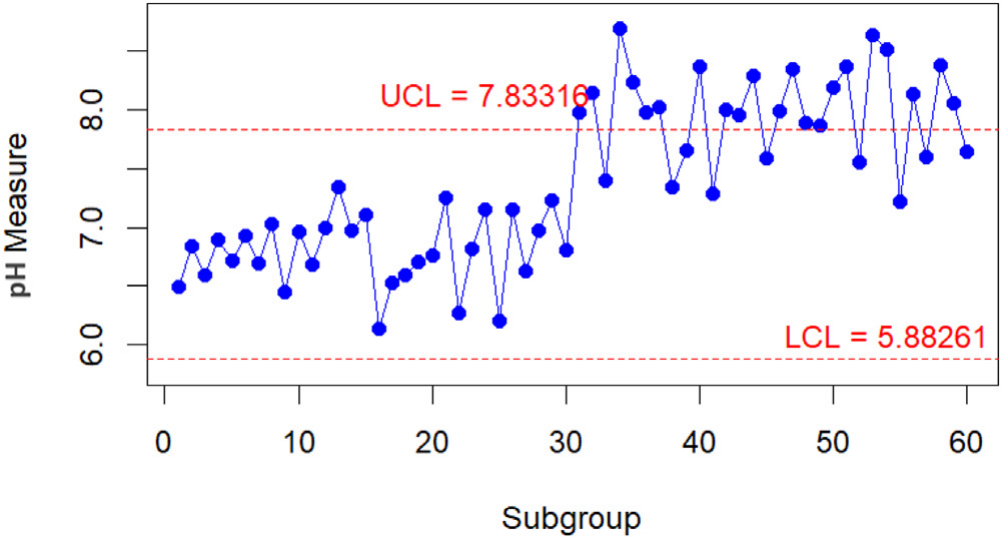
The improved Bajgier bootstrap Control Chart.

**Fig. 4:**
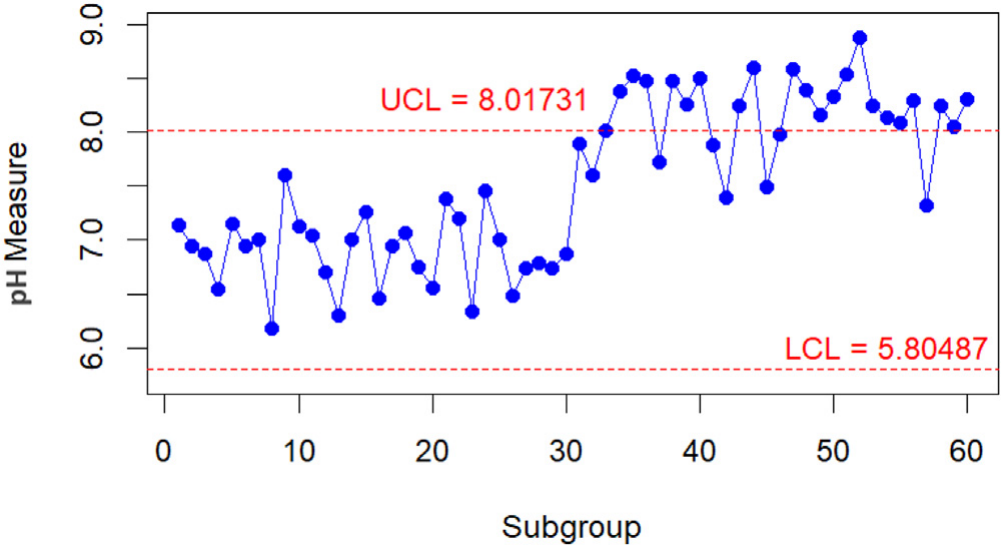
The improved Liu Tang bootstrap control chart.

To conduct a comparative analysis of the performance of the improved and classic bootstrap methods in their respective implementations. A practical scenario is presented when monitoring the pH value of water [23,24]. From the parameters taken from [23] for the water pH level, new data are generated into 30 subgroups, each with n=5 for both in-control and out-of-control conditions. The data with the in-control condition is assumed to come from a Gamma(70.13,0.098). In contrast, the data with the out-of-control condition is assumed to follow a Gamma(81.40,0.098). The results of the control chart for the Bajgier bootstrap control chart are illustrated in Fig. 1, the results of the control chart for the Liu Tang bootstrap control chart are illustrated in Fig. 2, the results of the control chart for the improved Bajgier bootstrap control chart are illustrated in Fig. 3, and the results of the control chart for the improved Liu Tang bootstrap control chart are illustrated in Fig. 4. The comparison results of the four control charts in monitoring data are also provided in Table 3.

**Table 3:** Comparison result.

Bootstrap Methods	The number of out of control detected
CBB	16
CLB	17
IBB	21
ILB	22

Based on the information from Table 3, which summarizes the performance of the four bootstrap control charts, it can be seen that the improved Liu Tang bootstrap control chart detected the most data in out-of-control conditions, with 22 out of 30 subgroups. This achievement was followed by the improved Bajgier bootstrap control chart, which detected 21 out of 30 subgroups. Meanwhile, the classic Bajgier and Liu Tang bootstrap control charts can only detect 16 and 17 out-of-control data points, respectively. From this information, the improved algorithm of the bootstrap control chart can enhance the ability to detect out-of-control data compared to the classic bootstrap control chart. This information is proven by the improved Bajgier bootstrap Control Chart, which can increase the ability to detect out-of-control data from 16 to 21 data points. Additionally, the improved Liu Tang bootstrap control chart can detect 22 data points compared to the classic Liu Tang bootstrap control chart, which can only detect 17 out-of-control data points. Therefore, based on the information presented, the improved bootstrap control chart with the improved algorithm performs better than the classic bootstrap control chart for both the Bajgier and the Liu Tang bootstrap control charts.

### Limitations

The performance measures used in this study only use AARL and SDARL. In addition, The data used to evaluate the improved bootstrap control chart were generated from Normal, Student's t and gamma distributions..

## CRediT authorship contribution statement

**Sukma Adi Perdana:** Conceptualization, Methodology, Software, Writing – original draft, Visualization. **Muhammad Mashuri:** Conceptualization, Methodology, Writing – review & editing, Validation, Supervision. **Muhammad Ahsan:** Conceptualization, Methodology, Writing – review & editing, Validation, Supervision.

## Declaration of competing interest

The authors declare that they have no known competing financial interests or personal relationships that could have appeared to influence the work reported in this paper.

## Data Availability

Data will be made available on request.
